# The evolving landscape of health professions education in Pakistan: A critical discourse on CHPE regulation and standardization

**DOI:** 10.12669/pjms.40.1.8783

**Published:** 2024

**Authors:** Syed Jaffar Abbas Zaidi, Syeda Rubaba Azim

**Affiliations:** 1 Dr. Syed Jaffar Abbas Zaidi, MD, RDS, MSc., MFDS RCPSG, MFD RCSI, FAIMER. Digital Learning Centre, Dow University of Health Sciences, Karachi, Pakistan; 2 Dr. Syeda Rubaba Azim, MBBS, MMed. Dow Institute of Health Professions Education, Dow University of Health Sciences, Karachi, Pakistan

The Certificate in Health Professions Education (CHPE) program, mandated by the Pakistan Medical and Dental Council (PMDC) for all medical and dental educators seeking promotion, serves as a pivotal development in the landscape of health professions education within Pakistan. Designed to be innovative, the CHPE program aims to furnish healthcare practitioners with the requisite educational proficiencies, knowledge base, and skill set to excel as educators, mentors, and leaders within the healthcare milieu. As the CHPE initiative undergoes broader implementation, concerns regarding its regulatory governance, standardization protocols, and adaptability to indigenous contexts have emerged, thus invoking the need for scholarly discourse.^1^ With the proliferation of medical universities in the country, there is a marked variability in the content, quality, and assessment criteria of CHPE programs across institutions. Furthermore, there is a trend of heavy reliance on Western medical education models without appropriate contextualization of Pakistan’s unique healthcare challenges and cultural intricacies. This inconsistency and potential lack of relevance emphasize the urgent need for standardized regulation to ensure the quality and applicability of these programs to address the country’s healthcare needs. This editorial undertakes an analytical exploration of the CHPE program, focusing on its efficacy, advantages, limitations, and the imperative for regulatory oversight.

## The Pros of the CHPE Program:

**1. Building Competency and Capacity:** The CHPE programs focus on building capacity in health education, pedagogical methodologies, and curriculum development. These programs elevate the quality of health education and create a sustainable health system.^2^
**2. Leadership Development:** This program prepares professionals for leadership roles in healthcare.
**3. Fostering Research Skills:** The program encourages an empirical approach to education, facilitating healthcare professionals to engage in scientific investigation of educational issues and to appraise educational interventions effectively.^3^

## The Cons of the CHPE Program:

**Fig.1:**
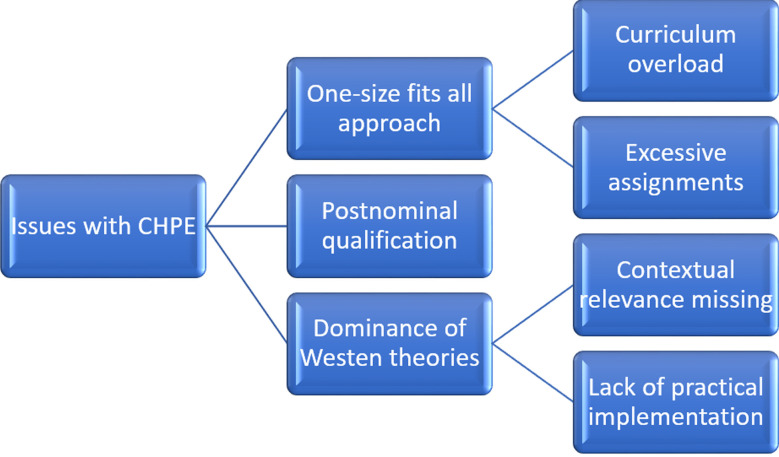
Issues with CHPE programs in Pakistan.

**1. One-size-fits-all approach:** Critics argue that CHPE programs often employ a generic curriculum, regardless of the contextual nuances. Given that most evidence in medical education is context-dependent, a one-size-fits-all curriculum may lack universal efficacy.
**2. Curriculum Overload:** There is a growing concern about curriculum overload, and some programs are going overboard with their content. There are CHPE programs that teach grant writing that may not be relevant to the responsibilities of medical educators. Such surplus content and stringent evaluation criteria may impede a balanced assessment of participants’ competencies.^4^ This lack of standardized guidelines may contribute to variable quality across different programs.
**3. Excessive Assessments:** Some CHPE programs have faced criticism for their strict evaluation methodologies, such as penalizing students for inadequately formulated reflective diaries, potentially fostering a high-stress educational environment, and detracting from effective learning. Conversely, some CHPE programs are too lenient.
**4. CHPE as a post-nominal qualification:** While the CHPE program serves a pivotal role in enhancing the educational proficiencies of healthcare practitioners, questions have been raised about the overconfidence exhibited by CHPE graduates and the misapplication of the certification title. This undue confidence can impede ongoing learning and adaptability, essential facets in the ever-evolving landscape of medical education. Moreover, there have been instances of inappropriate use of the CHPE designation as a post-nominal qualification, a practice inconsistent with Pakistan Medical and Dental Council (PMDC) regulations. Such misuse indicates a misunderstanding of the program’s objectives, as it is a certification intended to bolster competency in medical education rather than serve as a formal academic credential. A diagram illustrating these issues is shown in Fig.1.

## Need for Regulation and Standardization of CHPE Programs:

There is a strong demand for standardization and regulatory oversight of the CHPE programs to address the challenges. This approach entails reconceptualizing medical education in a more inclusive way with social sciences, where knowledge is not solely dichotomous or universally valid but context-specific and nuanced. The curriculum should be critically reviewed and streamlined to ensure it encompasses essential competencies without overburdening the participants. Standardization should be focused on achieving competency outcomes rather than rigid adherence to a particular content format.

Furthermore, assessment methods must be refined to ensure they are fair and educational, fostering a supportive environment conducive to learning and professional growth. However, striking a balance between maintaining standards and allowing flexibility for programs to cater to specific needs and contexts is crucial.

The PMDC, as the oversight body, should engage in active dialogue with educational institutions and health professionals to address these concerns to improve the overall quality of healthcare education in Pakistan.

## Recommendations

**Fig.2:**
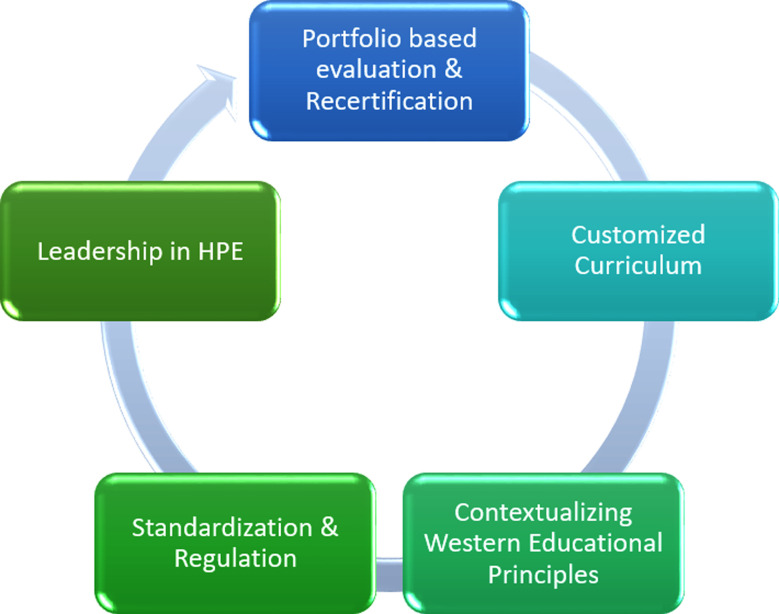
Recommendations for improving CHPE programs.

The following are possible solutions for the problems CHPE programs in Pakistan currently face, as illustrated in Fig.2.

## Overcoming Curriculum Overload:

A primary concern in current CHPE programs is curriculum overload. The diversity of content—ranging from direct medical education principles to tangential skills such as grant writing—presents a risk of diluting the focus and overwhelming learners.^5^

## Portfolio-Based Evaluation and Recertification for Quality Assurance:

Addressing the misunderstanding of CHPE’s purpose and the misuse of its credentials as a post-nominal qualification requires introducing comprehensive and continuous evaluation systems. A proposed portfolio-based evaluation system offers a promising solution. Candidates can maintain a record of their training, services, and program evaluation results, ensuring ongoing professional development and consistent contributions to medical education.^6^ Recertification would not only serve to maintain standards within the CHPE cadre but would also encourage a mindset of ongoing learning and adaptation among the certificate holders. It would reinforce the idea that mastery in medical education is an ongoing process, not a one-time achievement and that medical education requires continuous engagement with new knowledge, skills, and practices.^7^ This strategy could introduce an extra tier of quality control, thereby augmenting the CHPE program’s credibility and standing.

## Leadership in Health Professions Education:

Principals and deans play a significant role in the education of the health professions. By participating in the CHPE program, they can improve the quality of their decision-making and foster a culture of lifelong learning.^8^

The role of principals and deans in medical and dental schools extends far beyond administrative responsibilities. As leaders, their decisions significantly impact the education of health professions, shaping the direction of curricula, pedagogy, and the overall learning environment. Given their influence, it is worthwhile to propose that principals and deans undertake the Certificate in Health Professions Education (CHPE). In light of their substantial impact, suggesting that principals and deans also undertake CHPE certification is a meaningful proposition. Requiring principals and deans to undertake CHPE could have several potential benefits:

1. **Better Decision Making:** These leaders could make more informed and effective decisions based on a solid understanding of contemporary theory and practice.
2. **Leading by Example:** By undertaking the CHPE themselves, principals and deans set a precedent for the rest of the faculty, emphasizing the importance of continuous professional development.
3. **Enhanced Communication and Collaboration:** With a shared understanding and vocabulary of health professions education, principals, deans, and faculty can have more productive dialogues about improving education delivery.

However, making it mandatory for principals and deans to undertake the CHPE might also be met with some challenges but the potential benefits to health professions education make it a worthwhile consideration

1. **Time Constraints:** Principals and deans may have difficulty allocating enough time for CHPE programs due to their administrative duties.
2. **Need for Customized Content:** The standard CHPE program may not address the specific strategic and managerial needs of principals and deans in health professions education. An executive-focused CHPE program could be better suited for them.

## Contextualizing Western Educational Principles:

Edward Said, renowned for his work on postcolonial theory, viewed modernity predominantly as an extension of Westernization.^9^ His theory emphasizes the cultural and historical context in understanding any phenomenon, including education. Applying Said’s perspective on the modernization of medical education, specifically in the context of Pakistan’s CHPE programs, opens up a unique viewpoint.

Said’s theory of Orientalism could provide a framework for understanding the influence of Western principles on medical education in countries like Pakistan. Orientalism posits that the West’s perception and representation of the East are inherently biased, portraying Eastern societies as exotic, backward, uncivilized, and, at times, dangerous.^10^

Medical education has primarily been guided by Western principles and pedagogical strategies. However, when implementing CHPE programs in Pakistan, it is crucial to recognize that these Western principles might not always be contextually appropriate. Learning theories and learning strategies need to be taught and incorporated with caution, acknowledging their Western origins and carefully considering their suitability for Pakistani cultural, social, and economic contexts.

For instance, self-directed learning, a widely accepted strategy in Western education, might not resonate the same way in a Pakistani context where the traditional teaching methods are more didactic. Similarly, other learning theories, such as constructivism or problem-based learning, might require adaptations to integrate into the socio-cultural fabric of Pakistan effectively.

Therefore, CHPE programs in Pakistan should strive to balance modern, evidence-based educational strategies with local cultural norms and values. Contextualizing and adapting Western theories to local needs is more important than replicating them. A potential solution would be to involve local stakeholders -- educators, learners, administrators -- in curriculum development.

Said’s postcolonial theory emphasizes that modernizing medical education should not just adopt Western approaches but integrate both Western and local knowledge, considering the context’s significance in shaping effective educational strategies. The inclusion of local stakeholders in curriculum development and exploration of indigenous knowledge can contribute to a culturally sensitive, contextually relevant, and effective program.8

The Certificate in Health Professions Education (CHPE) program, under the PMDC’s purview, signifies a progressive step towards improving health professions education in Pakistan. The program has notable merits, including fostering competency and capacity in health education, cultivating leadership, and encouraging a research-oriented approach to health education. However, the implementation of the CHPE program is not without its challenges. There is a compelling call for regulating and standardizing these programs in alignment with the regional needs and contexts of different educational institutions.

The PMDC, as a regulatory body, should work in collaboration with educational institutions and health professionals to ensure that these programs are appropriately tailored to local needs. Streamlining the curriculum and refining assessment methods are potential starting points for making CHPE programs more contextually relevant and less burdensome for the participants.

Regulating and standardizing CHPE programs in Pakistan requires a careful and nuanced approach. This approach should respect the local context while leveraging global best practices, fostering a culture of continuous learning and development.

## Authors’ Contribution:

**SJAZ and RA** conceived, designed, and wrote the manuscript conjointly and are responsible for the integrity of the research.

**SJAZ and RA** reviewed and gave final approval of the manuscript.

## References

[1] Frenk J, Chen L, Bhutta ZA, Cohen J, Crisp N, Evans T (2010). Health professionals for a new century:transforming education to strengthen health systems in an interdependent world. Lancet.

[2] Togami E, Gardy JL, Hansen GR, Poste GH, Rizzo DM, Wilson ME (2018). Core competencies in one health education:what are we missing?. NAM Perspectives.

[3] Frank JR, Snell L, Englander R, Holmboe ES, Collaborators I (2017). Implementing competency-based medical education:Moving forward. Med Teach.

[4] Bordage G (1987). The curriculum:overloaded and too general?. Med Educ.

[5] Chen AM, Brown S, Mark K, McBane S (2023). An overview of Instructional approaches and decision-making strategies to curtail curricular OVERLOAD. Am J Pharm Educa.

[6] Driessen E, van Tartwijk J, Dornan T (2008). The self critical doctor:helping students become more reflective. BMJ.

[7] Irby DM, Cooke M, O'Brien BC (2010). Calls for reform of medical education by the Carnegie Foundation for the Advancement of Teaching:1910 and 2010. Acad Med.

[8] Tekian A, Roberts T, Batty HP, Cook DA, Norcini J (2014). Preparing leaders in health professions education. Med Teach.

[9] Dunch R (2002). Beyond cultural imperialism:Cultural theory, Christian missions, and global modernity. Hist Theory.

[10] Breckenridge CA, Van der Veer P (1993). Orientalism and the postcolonial predicament:Perspectives on South Asia;University of Pennsylvania Press.

